# Psychological stress and coeliac disease in childhood: a cohort study

**DOI:** 10.1186/1471-230X-10-106

**Published:** 2010-09-14

**Authors:** Karl Mårild, Anneli Sepa Frostell, Jonas F Ludvigsson

**Affiliations:** 1 Astrid Lindgren Children's Hospital, Karolinska University Hospital, Solna, Sweden; 2 Department of Medicine, Örebro University, Örebro, Sweden; 3 Department of Clinical and Experimental Medicine, Division of Paediatrics and Diabetes Research Centre, Linköping University, Sweden; 4 Clinical Epidemiology Unit, Department of Medicine, Karolinska Institutet, Stockholm, Sweden; 5 Department of Paediatrics, Örebro University Hospital, Örebro, Sweden

## Abstract

**Background:**

Psychological stress has previously been associated with several immunological diseases, e.g. inflammatory bowel disease. Through questionnaire data from the ABIS study (All Babies In southeast Sweden) we examined the association between psychological stress in the family and biopsy-proven coeliac disease (CD) in the child.

**Methods:**

We used serious life event, parenting stress, and parental worries as measures of psychological stress. Data were collected when the child was 1 and 2.5 years old in some 11,000 and 8,800 children, respectively. CD was confirmed through small intestinal biopsy (with villous atrophy), and the diagnosis was validated through patient chart data.

**Results:**

Serious life event in the family in the child's first 1 or 2.5 years after childbirth was not associated with future CD in the child (Odds Ratio (OR) = 0.45; 95% Confidence Interval (CI) = 0.01-2.65; *P *= 0.72; and OR = 1.21; 95% CI = 0.43-3.05; *P *= 0.64, respectively). Neither did we see any association between CD and parenting stress at age 1 year and at 2.5 years (OR = 0.40; 95% CI = 0.01-2.38; *P *= 0.73 and OR = 0.74; 95% CI = 0.01-4.56; *P *= 1.00, respectively). Among children exposed to parental worries at 2.5 years, no child had a diagnosis of CD before end of follow-up, compared to 25/8082 (0.3%) among non-exposed children (OR = 0.00; 95% CI = 0.00-2.34; *P *= 0.64). There was no association between the combined measures of stress and CD.

**Conclusion:**

This study found no association between psychological stress and later development of CD in Swedish children. However, we cannot rule out that the lack of such an association is due to limited statistical power.

## Background

Coeliac disease (CD) is an immunological disorder induced by gluten exposure. It is a multi-factorial disease characterized by small intestinal villous atrophy [1]. Previous studies have suggested that environmental factors in early life influence the risk of CD, e.g. the amount [2] and timing of gluten introduction [3]. It has been proposed that the first period of life may include a vulnerable timeframe, where environmental factors could influence the immune system and the risk of CD in childhood [4]. The proportion of CD cases identified through screening has increased during the last decades. This suggests that still unknown environmental factors remain to be identified [5].

Psychological stress is an environmental factor that has been associated with several immunological diseases, e.g. inflammatory bowel disease [6], allergic disease [7], and atopic dermatitis [8]. Psychological stress has a direct effect on a variety of immunological mechanisms, including the functional profile of T-cells and several immune-inflammatory markers [9,10].

In 2005, Sepa et al [11] suggested that maternal exposure to serious life events after childbirth increases the risk of diabetes-related autoimmunity in the offspring. Type 1 diabetes mellitus (T1D) and CD are closely associated [12], and the patients share similar HLA alleles, epidemiological features [13] and partially common infant feeding risk factors [3,14,15]. However an earlier study of pregnancy-related stress found no relationship between psychological stress in mothers *during pregnancy *and development of CD in the offspring [16]. In the current study, we examined the risk of CD in children to parents with high level of stress *after birth*. For this purpose we examined prospectively collected data on psychological stress in 73 children with biopsy-verified CD. This study was part of the ABIS project (All Babies in Southeast Sweden).

## Methods

### Description of the ABIS study

From October 1997 through October 1999, parents to babies born in southeast Sweden were invited to participate in the ABIS cohort project. This project examines the role of environmental factors for the development of autoimmune and allergic diseases. Of the 21,700 babies born during the study period, the parents of 17,055 children (78.6%) gave their informed consent to participate. In the maternity ward, the mothers received an at-birth questionnaire that was completed by 16,070 mothers. Of the 10,932 that participated in the 2.5-year follow-up, 8,805 completed the 2.5-years-questionnarie. Study participants [17] were more often born in Sweden, compared to the general population. We compared prospectively assessed measures of stress between children with CD with those without a diagnosis of CD.

### Definition of CD

Data on CD were collected on two occasions. The majority of children with CD were identified through a study on symptoms and signs in CD [18]. In 2007-2008, we again contacted the same eight paediatric departments participating in the study published in 2004 [18] and asked them to report additional ABIS children with biopsy-verified CD (partial or subtotal-total villous atrophy) diagnosed before 1^st ^Dec 2006. For this diagnosis we also requested symptoms/signs and antibody markers consistent with the diagnosis of CD (for further details, see earlier study [18]). In the current study, date of CD diagnosis equals date of first positive small intestinal biopsy. The ABIS child population was not actively screened for CD, and we do not have population-based data on CD serology in study participants. Hence, cases of CD were diagnosed due to symptoms, and signs, or through screening for clinical (i.e. non-research) purposes.

We identified 83 children with CD, 10 of these were diagnosed before age 1 year and excluded from the analyses since the diagnosis per se might have influenced the way the parents responded to the questions about stress and life events occurring within the first year of life. The remaining 73 were included in all analyses referring to stress until age 1 year, of these 43 were diagnosed after 2.5 year of age and were also included in the analyses referring to stress until age 2.5 year.

### Definition of psychological stress

Three domains concerning psychological stress were assessed: **1) Exposure to a serious life event **in the family was assessed at 1 and 2.5 years of age with the following two yes/no questions: At 1 year of age we asked: "Has your child been exposed to something which you perceive as a serious life event since his/her birth?". At 2.5 years of age we asked: "Have you [the parent] been exposed to something which you perceive as a serious life event since you're child's birth?". Examples given were death of a relative, serious disease in the family, serious accident in the family, divorce, exposure to violence, and unemployment. We had data on serious life event in 10,541 children (447; 4.2% had been exposed) in the first year of life, and in 8,722 children until age 2.5 years (2,119; 24.3% exposed). **2) Parenting stress **was assessed at 1 and 2.5 years of age with the Swedish Parenting Stress Questionnaire (SPSQ) [19], which has a good validity and good stability [19]. In our sample Chronbach's alpha was 0.88 at age 1 and 0.89 at age 2.5. SPSQ consists of 34 items tapping the dimensions incompetence (11 items), spouse relationship problems (5 items), role restriction (7 items), social isolation (7 items), and health problems (4 items). On each item a 6-point Likert-type response scale was used ranging from "strongly disagree" to "strongly agree". A mean value was calculated if less than six items were missing. Dichotomized variables were created using the 95^th ^percentile as a cut-off, defining exposure to parenting stress at each age. **3) Parental worries **were assessed at 2.5 years of age with six items, each describing a potential risk for the child (i.e. that the child falls seriously ill, is harmed, is going to be handicapped, is not going to develop normally, is going to be exposed to abuse, and is not going to survive). For each item the parent estimated on a 6-point Likert-type response scale ranging from "very calm" to "very worried" how worried they were that their child might become affected. Chronbach's alpha was 0.89 in our sample. Mean values for answered items (if one or no item was missing) above the 95^th ^percentile defined exposure to parental worries.

Finally, a **composite measure of psychological stress at age 2.5 **was created (from 8,369 children) in order to estimate the overall amount of stress experienced in the family, by counting in how many of the domains the child had been exposed to stress at age 2.5 (composite measures of this kind have been used in e.g. the papers by Ostberg et al [20] and Wekerle et al [21]). If a child had not been exposed in any of the domains the score for the composite measure was 0 and if the child had been exposed in all three domains the score was 3. Since only 11 children had been exposed in all three domains, they were grouped together with those exposed in two domains. This group will onwards be referred to as children exposed to high stress in the family.

### Statistics

To avoid potential recall bias, we excluded individuals with a diagnosis of CD before the age of 1 year from our main analyses (since knowledge of CD in one's child might have influenced the way the questionnaires were completed by parents) (and before 2.5 years of age in relevant analyses). Hence follow-up began at 1 year of age.

Chi-2 test, and when appropriate Fisher' exact test, and logistic regression estimated the association between the psychological stress variables and CD. Exact odds ratios (OR) and 95% confidence intervals (CI) were calculated to compare children 'exposed' and 'not exposed' concerning each psychological stress domain, respectively, as well as for comparing 'not exposed', 'exposed in one domain', and 'high stress in the family' (i.e. exposed in at least two domains) for the composite measure. The dependent variables in the analyses were CD after age 1 year (*n *= 73), CD after age 2.5 years (*n *= 43), and ever having a diagnosis of CD (*n *= 83). Due to lack of data on stress (not all study participants filled out the questionnaires at 1 year and 2.5 years of age or all questions in the questionnaires), the actual number of children with CD included in the analyses was lower (1 year: n = 48-51; 2.5 year: n = 24-26).

Since earlier data have suggested that breastfeeding pattern may influence the risk of CD [2], we chose to adjust for breastfeeding duration in a number of post-hoc analyses. We had data on duration of breastfeeding in 8,428 individuals. Children were divided into four categories (≤ 90 days, 91-180 days, 181-270 days and ≥ 271 days of breastfeeding). In individual analyses, numbers may be lower due to missing data on outcome measures.

Statistical significance was defined as 95% CI for estimates not including 1.0 *and *p < 0.05. We used SPSS 15.0 to perform the analyses.

### Ethics

This study was part of the ABIS study, which has been approved by the Research Ethics Committees of the Faculty of Health Sciences, Linkoping University, and the Medical Faculty of Lund University. Mothers gave their consent after careful written as well as oral information and information via videotape.

## Results

### Background data

More than 70% of patients with CD were girls compared to about 48% in those without a diagnosis of CD (see Additional file 1). A family history of T1 D and CD was more common among children with CD compared to the reference group. The children were followed-up until Dec 1^st ^2006, which corresponded to 8 years of age in the average participant. The mean maternal age at childbirth was close to 30 years.

### Serious life event

Out of 10,541 children, 447 had experienced at least one serious life event during their first year of life according to questionnaire data (Table 1). Only 1/447 exposed to a serious life event developed CD. This corresponded to an OR for future CD of 0.45 (95% CI = 0.01-2.65; *P *= 0.72). At 2.5 years of life 2,119/8,722 reported that they had experienced a least one serious life event (Table 2). Seven children whose parent had experienced a serious life event had a later diagnosis of CD (0.33%), compared to 18 (0.27%) children without a parental experience of serious life event. Serious life event in the first 2.5 years of life was hence no risk factor for future CD (OR = 1.21; 95% CI = 0.43-3.05; *P *= 0.64).

**Table 1:** Psychological stress at age 1 and risk of future coeliac disease (CD).

	*Crude odds ratios (OR) for coeliac disease* *diagnosed after age 1*				
	n *	***n *with CD**	**OR**	**95% CI**	***P***

**Serious life event at age 1 **§					0.72

No serious life event	10094	50	reference		

A Serious life event	447	1	0.45	0.01-2.65	

					

**Parenting stress at age 1**§					0.73

Non-exposed	9989	47	reference		

Exposed	525	1	0.40	0.01-2.38	

*Number of parents answering the questionnaire questions referring to serious life event and parenting stress, respectively. Due to internal attrition the numbers of reference individuals vary between the different analyses.

§For definition of serious life event and parenting stress, see text.

CI = Confidence interval according to exact test. P-values were calculated using Fisher's exact test.

**Table 2:** Psychological stress at age 2.5 years and risk of future coeliac disease (CD).

	*Crude odds ratios (OR) for coeliac disease diagnosed after age 2.5*				
	n*	***n *with CD**	**OR**	**95% CI**	***P***

**Serious life event at age 2.5 **§					0.64

No serious life event	6603	18	reference		

A Serious life event	2119	7	1.21	0.43-3.05	

					

**Parenting stress at age 2.5 **§					1.00

Non-exposed	8202	25	reference		

Exposed	442	1	0.74	0.01-4.56	

					

**Parental worries at age 2.5 **§					0.64

Non-exposed	8082	25	reference		

Exposed	438	0	0.00	0.00-2.34	

					

**Composite measure at age 2.5 **§					

Non-exposed	5837	18	reference		

Exposed in one domain	2232	5	0.73	0.21-2.03	0.64

High stress in the family	300	1	1.08	0.03-6.89	0.61

*Number of parents answering the questionnaire questions referring to serious life event, parenting stress and parental worries, respectively. Due to internal attrition the numbers of reference individual vary between the different analyses.

§For definition of serious life event, parenting stress, parenting worries and the composite measure for stress, see text.

CI = Confidence interval according to exact test. P-values were calculated using Fisher's exact test.

### Parenting stress

Only 1/525 children exposed to such stress developed CD (0.19%) (compared with 47 non-exposed children who developed CD (0.47%); Table 1). OR for CD in children exposed to parenting stress at age 1 was 0.40 (95% CI = 0.01-2.38; *P *= 0.73). The corresponding OR at 2.5 years was 0.74 (95% CI = 0.01-4.56; *P *= 1.00) (Table 2).

### Parental worries

In 438 children (out of 8,520), parents had reported worries in the first 2.5 years of life. None of these children later developed CD (compared with 25 non-exposed children). CD was hence not associated with parental worries during the first 2.5 year of life (Table 2). When we included all individuals with CD, also those with a diagnosis prior to the 1-year-questionnaire there was still no association between parental worries and CD (OR = 0.80; 95% CI = 0.09-3.08; *P *= 1.00).

### Composite measure of psychological stress

In a final analysis we used logistic regression to estimate the association between a composite measure of psychological stress across three different domains (serious life event, parenting stress and parental worries) and CD. However, CD was not associated with stress in one or more domains (Table 2).

### Post-hoc analyses adjusting for duration of breastfeeding

Adjustment for duration of breastfeeding did not influence any of the above risk estimates more than marginally (complete data not shown). E.g. there were no increased risk of future CD in individuals with a serious life event at age 1 (adjusted OR = 0.61; 95% CI = 0.08-4.47; *P *= 0.63), parenting stress at age 1 (adjusted OR = 0.54; 95% CI = 0.07-3.94; *P *= 0.54), or composite measure of high stress in the family at age 2.5 (adjusted OR = 1.35; 95% CI = 0.18-10.26; *P *= 0.77).

## Discussion

In the current study we examined the association between psychological stress in the family (measured as serious life event, parenting stress, and parental worries, respectively and combined), and biopsy-proven CD in the child. There was no statistically significant association between prospectively assessed exposure to psychological stress in the family and later development of CD in the child. However, due to small numbers we cannot rule out that early infant stress may nevertheless considerably influence the risk of CD.

A major strength of our population-based study is its prospective design, where data on stress were collected prior to the diagnosis of CD. This fact eliminates the risk of recall bias dependent on CD status. The CD diagnosis was based on small intestinal biopsy (with villous atrophy) and validated through review of patient chart data supplied from the responsible physicians at each of the eight paediatric departments caring for CD in the study area. Hence, the risk of misclassification is low. Another strength is our use of a validated scale to measure parenting stress (Swedish Parenthood Stress Questionnaire (SPSQ)) [19].

Similar to most other prospective cohort studies, the study suffers from some drop-out. At 2.5 years of age, half of the population entering the study at birth completed the relevant questionnaire. Although, we cannot rule out the existence of bias, our study design guarantees that such potential bias is independent of CD status. It has been shown that the participants in the ABIS study cohort have a slightly lower proportion of mothers with foreign origin and a slight under representation of parents with low education compared to the general Swedish population [11].

Children in this study were on average followed up until 8 years of age. It is likely that some individuals have a diagnosis of CD after that age, but given the prevalence of CD in our dataset, we assume that a large share of individuals with diagnosed CD will have received their diagnosis before age 9 years. And there is little reason to believe that parental stress in the first 1-2 years of life will not affect the risk of CD in the first 8 years, but well beyond that age. Of greater concern is that we have not been able to screen the ABIS cohort for CD, and our patients only consist of individuals with diagnosed CD. That is however only a problem if risk factors differ between individuals with or without symptoms, and individuals with or without high disease activity (meriting investigation for CD in childhood). Although, we cannot rule out that the inclusion of undiagnosed CD would have altered our risk estimate; psychological stress in the family is unlikely to be a major risk factor for CD, since it does not influence the risk of diagnosed CD at all (see results section). Meanwhile, the inclusion of false-negative CD cases among controls in this study will not affect risk estimates since the prevalence of undiagnosed CD should not exceed 1% [22].

One weakness of this study is the lack of means to directly measure the psychological stress in the *child*. Instead this study, like earlier research [11,17], used psychological stress in the *family*, assessed by the parent, as a marker for stress in the child. Although psychological stress in the family is most likely to influence the child's wellbeing and experience of stress, we cannot rule out that this indirect measure of psychological stress have changed our risk estimates and increased the risk of a type 2 error. Due to internal attrition the actual number of children with CD included in the analyses was low (1 year: n = 48-51; 2.5 year: n = 24-26), which also contributed to an increased risk of a type 2 error (i.e. to erroneously accept a false null hypothesis).

In a negative trial, like this one, the clinical significance of a potential type 2 error must be remembered. We cannot rule out that we have failed to observe a difference in the risk of CD between exposed and unexposed children due to lack of study power. Some of the summary risk estimates in this study are below one and, although far from statistical significant, may even indicate a negative association (i.e. a *protective *effect) between psychological stress and CD. This study cannot rule out that non-exposed children are at a highly increased relative risk of future CD, however, the baseline prevalence of CD in this group was still only 0.31%.

Data on the protective effect of breastfeeding on the risk of CD are conflicting [2,3,23]. Earlier data on the ABIS children [24] have shown no association between duration of breastfeeding and CD status (See Methods). In a number of post-hoc analyses we still chose to adjust for duration of breastfeeding, but with only marginally changed risk estimates. Low socioeconomic status (e.g. level of education) and maternal age have been suggested to be associated with parenting stress [25]. However, in this study there was no significant difference in maternal age or level of education according to CD status (univariate analyses) hence they we did not adjust for these values [26].

Earlier data suggest a positive association between psychological stress and a number of diseases [27,28] including T1D-related autoimmunity [29]. T1 D shares many etiological traits with CD [30] including shared infant feeding risk factors [3,14,15], occurrence of similar autoantibodies [12] and a shared genetic susceptibility [31]. CD and T1 D also share certain epidemiological features, and the prevalence of CD among patients with T1 D is estimated to be in the range of 3-6% [22]. Using prospectively collected data from the ABIS study, Sepa et al [11,17] found an association between mothers' experience of psychological stress and diabetes-related *autoimmunity *in their children in infancy and at age 2.5 years. Based on other data from the ABIS study, Ludvigsson and Ludvigsson failed to show any association between pregnancy-related stress in the mother and risk of CD in the offspring [16].

## Conclusions

The results of the current study are consistent with our earlier data on stress and CD, and we found no statistically significant association between psychological stress in the family in the first 2.5 years of life and future CD in childhood. However, we cannot rule out that the lack of such an association is due to limited statistical power.

## List of abbreviations

ABIS: all Babies in Southeast Sweden; CD: coeliac disease; CI: confidence interval; OR: odds ratio; SPSQ: Swedish parenting stress questionnaire; TID: type 1 diabetes mellitus.

## Competing interests

The authors declare that they have no competing interests.

## Authors' contributions

KM wrote the manuscript and contributed to the study design. AF contributed with knowledge on stress and the instruments to measure stress, she also performed the statistical analyses. JFL initiated and designed the study. AF and JFL critical reviewed the manuscript. All authors read and approved the final manuscript.

## Pre-publication history

The pre-publication history for this paper can be accessed here:

http://www.biomedcentral.com/1471-230X/10/106/prepub

## Supplementary Material

Additional file 1 **Background characteristics of the ABIS study cohort according to presence of future coeliac disease (CD)**. Click here for file

## References

[B1] Green PH Cellier C Celiac disease N Engl J Med 2007 357 1731 1743 10.1056/NEJMra071600 17960014

[B2] Ivarsson A Hernell O Stenlund H Persson LA Breast-feeding protects against celiac disease Am J Clin Nutr 2002 75 914 921 11976167 10.1093/ajcn/75.5.914

[B3] Norris JM Barriga K Hoffenberg EJ Taki I Miao D Haas JE Emery LM Sokol RJ Erlich HA Eisenbarth GS Rewers M Risk of celiac disease autoimmunity and timing of gluten introduction in the diet of infants at increased risk of disease Jama 2005 293 2343 2351 10.1001/jama.293.19.2343 15900004

[B4] Guandalini S Setty M Celiac disease Curr Opin Gastroenterol 2008 24 707 712 10.1097/MOG.0b013e32830f4527 19122520

[B5] Lohi S Mustalahti K Kaukinen K Laurila K Collin P Rissanen H Lohi O Bravi E Gasparin M Reunanen A Maki M Increasing prevalence of coeliac disease over time Aliment Pharmacol Ther 2007 26 1217 1225 10.1111/j.1365-2036.2007.03502.x 17944736

[B6] Mawdsley JE Rampton DS Psychological stress in IBD: new insights into pathogenic and therapeutic implications Gut 2005 54 1481 1491 10.1136/gut.2005.064261 16162953 PMC1774724

[B7] Montoro J Mullol J Jauregui I Davila I Ferrer M Bartra J del Cuvillo A Sastre J Valero A Stress and allergy J Investig Allergol Clin Immunol 2009 19 Suppl 1 40 47 19476053

[B8] Arndt J Smith N Tausk F Stress and atopic dermatitis Curr Allergy Asthma Rep 2008 8 312 317 10.1007/s11882-008-0050-6 18606083

[B9] Marshall GD Jr Agarwal SK Lloyd C Cohen L Henninger EM Morris GJ Cytokine dysregulation associated with exam stress in healthy medical students Brain Behav Immun 1998 12 297 307 10.1006/brbi.1998.0537 10080859

[B10] Glaser R MacCallum RC Laskowski BF Malarkey WB Sheridan JF Kiecolt-Glaser JK Evidence for a shift in the Th-1 to Th-2 cytokine response associated with chronic stress and aging J Gerontol A Biol Sci Med Sci 2001 56 M477 482 11487599 10.1093/gerona/56.8.m477

[B11] Sepa A Frodi A Ludvigsson J Mothers' experiences of serious life events increase the risk of diabetes-related autoimmunity in their children Diabetes Care 2005 28 2394 2399 10.2337/diacare.28.10.2394 16186269

[B12] Ascher H Coeliac disease and type 1 diabetes: an affair still with much hidden behind the veil Acta Paediatr 2001 90 1217 1220 10.1080/080352501317130209 11808887

[B13] Bao F Yu L Babu S Wang T Hoffenberg EJ Rewers M Eisenbarth GS One third of HLA DQ2 homozygous patients with type 1 diabetes express celiac disease-associated transglutaminase autoantibodies J Autoimmun 1999 13 143 148 10.1006/jaut.1999.0303 10441179

[B14] Norris JM Barriga K Klingensmith G Hoffman M Eisenbarth GS Erlich HA Rewers M Timing of initial cereal exposure in infancy and risk of islet autoimmunity JAMA 2003 290 1713 1720 10.1001/jama.290.13.1713 14519705

[B15] Ziegler AG Schmid S Huber D Hummel M Bonifacio E Early infant feeding and risk of developing type 1 diabetes-associated autoantibodies Jama 2003 290 1721 1728 10.1001/jama.290.13.1721 14519706

[B16] Ludvigsson JF Ludvigsson J Stressful life events, social support and confidence in the pregnant woman and risk of coeliac disease in the offspring Scand J Gastroenterol 2003 38 516 521 12795462

[B17] Sepa A Wahlberg J Vaarala O Frodi A Ludvigsson J Psychological stress may induce diabetes-related autoimmunity in infancy Diabetes Care 2005 28 290 295 10.2337/diacare.28.2.290 15677781

[B18] Ludvigsson JF Ansved P Falth-Magnusson K Hammersjo JA Johansson C Edvardsson S Ljungkrantz M Stenhammar L Ludvigsson J Symptoms and Signs Have Changed in Swedish Children With Coeliac Disease J Pediatr Gastroenterol Nutr 2004 38 181 186 10.1097/00005176-200402000-00015 14734881

[B19] Ostberg M Hagekull B Wettergren S A measure of parental stress in mothers with small children: dimensionality, stability and validity Scand J Psychol 1997 38 199 208 10.1111/1467-9450.00028 9309950

[B20] Ostberg M Parental stress, psychosocial problems and responsiveness in help-seeking parents with small (2-45 months old) children Acta Paediatr 1998 87 69 76 10.1080/08035259850157903 9510451

[B21] Wekerle C Wall AM Leung E Trocme N Cumulative stress and substantiated maltreatment: the importance of caregiver vulnerability and adult partner violence Child Abuse Negl 2007 31 427 443 10.1016/j.chiabu.2007.03.001 17445887

[B22] Dube C Rostom A Sy R Cranney A Saloojee N Garritty C Sampson M Zhang L Yazdi F Mamaladze V Pan I Macneil J Mack D Patel D Moher D The prevalence of celiac disease in average-risk and at-risk Western European populations: a systematic review Gastroenterology 2005 128 S57 67 10.1053/j.gastro.2005.02.014 15825128

[B23] Peters U Schneeweiss S Trautwein EA Erbersdobler HF A case-control study of the effect of infant feeding on celiac disease Ann Nutr Metab 2001 45 135 142 10.1159/000046720 11463995

[B24] Welander A Tjernberg AR Montgomery SM Ludvigsson J Ludvigsson JF Infectious Disease and Risk of Later Celiac Disease in Childhood Pediatrics PMID: 20176673. Epub Feb 22, 2010 20176673 10.1542/peds.2009-1200

[B25] Leigh B Milgrom J Risk factors for antenatal depression, postnatal depression and parenting stress BMC Psychiatry 2008 8 24 10.1186/1471-244X-8-24 18412979 PMC2375874

[B26] Rothman K Greenland S Modern Epidemiology 1998 2 Philadelphia: Lippincott-Raven Publishers

[B27] Graham NM Douglas RM Ryan P Stress and acute respiratory infection Am J Epidemiol 1986 124 389 401 3740039 10.1093/oxfordjournals.aje.a114409

[B28] Boscarino JA Diseases among men 20 years after exposure to severe stress: implications for clinical research and medical care Psychosom Med 1997 59 605 614 9407579 10.1097/00006842-199711000-00008

[B29] Sepa A Ludvigsson J Psychological stress and the risk of diabetes-related autoimmunity: a review article Neuroimmunomodulation 2006 13 301 308 10.1159/000104858 17709952

[B30] Cronin CC Shanahan F Insulin-dependent diabetes mellitus and coeliac disease Lancet 1997 349 1096 1097 10.1016/S0140-6736(96)09153-2 9107261

[B31] Smyth DJ Plagnol V Walker NM Cooper JD Downes K Yang JH Howson JM Stevens H McManus R Wijmenga C Heap GA Dubois PC Clayton DG Hunt KA van Heel DA Todd JA Shared and distinct genetic variants in type 1 diabetes and celiac disease N Engl J Med 2008 359 2767 2777 10.1056/NEJMoa0807917 19073967 PMC2840835

