# Supplementary material for: Psychological stress and coeliac disease in childhood: a cohort study
Source: BMC Gastroenterol. 2010 Sep 14;10:106. doi: 10.1186/1471-230X-10-106 (PMC2945988; doi:10.1186/1471-230X-10-106)
Supplement: Additional file 1 — Background characteristics of the ABIS study cohort according to presence of future coeliac disease (CD). [file 1471-230X-10-106-S1.PDF]

Additional file

Additional file. Background characteristics of the ABIS study cohort according to presence of future coeliac disease (CD).

		Diagnosed after 1 year			*Diagnosed after 2.5 years*	
	Reference	CD		Reference	CD	
**Background variables assessed at birth**	*n* =10,742-11,029 §	*n*=70-73*(%)	*p*	*n* = 8,441-8,779 §	*n*=41-43* (%)	*p*
Female sex; *n* (%)	5264 (47.8)	53 (72.6)		4196 (48.0)	32 (74.4)	
Male sex; *n* (%)	5739 (52.2)	20 (27.4)	<0.001	4538 (52.0)	11 (25.6)	0.001
Age in months at the follow-up; mean (SD)	12.01 (1.17)	12.49 (1.68)	0.047	33.09 (3.590)	32.44 (5.100)	0.533
Heredity						
First-degree relative with CD; *n* (%)	149 (1.4%)	4 (5.7%)	0.016	126 (1.4)	0 (0)	*NA #*
First-degree relative with type 1 diabetes; *n* (%)	266 (2.4%)	9 (12.9%)	<0.001	207 (2.4)	6 (14.3)	<0.001
Demographics						
Maternal education >12 years; *n* (%)	3566 (33.2)	27 (39.1)	0.297	2904 (34.1)	17 (41.5)	0.320
Maternal age; mean (SD)	29.78 (4.542)	28.99 (4.185)	0.138	29.85(4.498)	29.12 (4.049)	0.245
Maternal age, ≥30 years; *n* (%)	5533 (50.3)	31 (42.5)	0.180	4383 (50.5)	20 (46.5)	0.606
Maternal origin, born outside Sweden; *n* (%)	671 (5.7)	1 (1.4)	0.188	457 (5.4)	0(0)	0.170

§ Due to internal attrition (not all participants completed the questionnaires at 1 year and at 2.5 years) the numbers of reference individuals vary between the different analyses.

* The following data were obtained from the birth questionnaire: sex of the infant, heredity for CD, type 1 diabetes, and maternal origin. Parents to 70 children diagnosed with CD after 1 year of age responded to the birth questionnaire; and parents to 42 children diagnosed after age 2.5 years. In one child with CD, the parents failed to complete the question on maternal educational (giving data available in 69 and 41 children with a future CD diagnosis for that variable).

# NA: Not applicable (statistical analysis was not possible to calculate).
